# Collective behavior diverges independently of the benthic-limnetic axis in stickleback

**DOI:** 10.1007/s00265-025-03599-z

**Published:** 2025-05-08

**Authors:** Kevin M. Neumann, Lucas Eckert, Damaris Miranda, Andrew Kemp, Alison M. Bell

**Affiliations:** 1 Program in Ecology, Evolution, and Conservation Biology, University of Illinois Urbana-Champaign, Urbana, IL USA; 2 Department of Biology and Redpath Museum, McGill University, Montréal, Québec Canada; 3 Department of Evolution, Ecology and Behavior, School of Integrative Biology, University of Illinois at Urbana-Champaign, Urbana, IL USA; 4 Carl R. Woese Institute for Genomic Biology, University of Illinois Urbana-Champaign, Urbana, IL USA; 5 Program in Neuroscience, University of Illinois Urbana-Champaign, Urbana, IL USA

**Keywords:** Social network, Boldness, *Gasterosteus aculeatus*, Behavioral syndromes, Parallel evolution, Population divergence

## Abstract

Comparing populations across replicate environments or habitat types can help us understand the role of ecology in evolutionary processes. If similar phenotypes are favored in similar environments, parallel evolution may occur. Collective behavior, including collective movement and social networks, can play a key role in the adaptation by animals to different environments. However, studies exploring the parallelism of collective behavior are limited, with research traditionally focusing on morphological traits. Here, we asked if collective behavior varies consistently across replicate populations of benthic and limnetic three-spined stickleback (*Gasterosteus aculeatus*). There were repeatable, population-level differences in collective behavior in a common garden experiment, with some populations forming groups that were more cohesive and with higher strength and clustering coefficients. However, these differences were not predicted by ecotype (benthic vs. limnetic). Latency to emerge and morphology did consistently differ between ecotypes, and there were no correlations between these traits and collective behavior. Together, these results suggest that collective behavior diverges, but not in a way that is associated with variation along the benthic-limnetic axis. By examining multiple traits, we show how phenotypic evolution can be occurring in parallel and non-parallel ways at the same time. This study further highlights that the classification of habitat types may only be relevant for certain traits, with collective behavior potentially changing in a more nuanced manner.

**Supplementary Information:**

The online version contains supplementary material available at 10.1007/s00265-025-03599-z.

## Significance statement

When animals are exposed to different environments, they may develop adaptations that are best fit for their local conditions. Understanding the effect of different environments on various traits including morphology and behavior can help us predict how future environmental changes might impact a species. Here, we compared shoaling behavior and social networks across eight populations of three-spined stickleback fish that tend to occur either in the open water or in the benthos (lake bottom) of lakes. We found that shoaling and social networks were heritable and repeatable, and differed across populations. However, these differences were not predicted by the habitat that the population was from. These findings suggest that behavior may evolve in a more nuanced manner relative to other traits associated with the benthic-limnetic axis. Future studies should explore other ecological conditions beyond this axis that might be driving these behavioral differences.

**Supplementary Information:**

The online version contains supplementary material available at 10.1007/s00265-025-03599-z.

## Introduction

Organisms often contend with different environments across their geographic range. If multiple populations originating from a common source population face similar selective pressures in their current environments, these populations may evolve similar phenotypes or genotypes (‘parallel evolution’; Clarke 1975; Orr 2005). A powerful way to test for parallel evolution is to compare the phenotypes of replicate populations of the same species from different environments or habitat types. Some classic examples include the reduction of armor when three-spined stickleback (*Gasterosteus aculeatus*; hereafter synonymous with ‘stickleback’) move from marine to freshwater (Colosimo et al. 2005), shifts in life history traits between high- and low-predation environments in guppies (*Poecilia reticulata*) (Reznick et al. 1996), and divergence in morphology as a function of microhabitat in *Anolis* lizards (Losos et al. 1998). Parallel evolution provides support for the power of natural selection and adaptation in shaping evolutionary outcomes. If parallel adaptations arise independently across populations exposed to similar selective pressures, we could use this knowledge to predict future evolutionary change (Agrawal 2017).

Empirical studies of parallel evolution in animals often focus on morphological traits (Oke et al. 2017; Bolnick et al. 2018), but behavioral traits can also exhibit parallel evolution (Ingley et al. 2014; York and Fernald 2017). For instance, nine-spined stickleback (*Pungitius pungitius*) in low predation environments were consistently bolder and more aggressive than those in high predation environments (Herczeg et al. 2009), and *Heliconius* butterflies exhibit higher activity levels at higher elevations (Rivas-Sanchez et al. 2023). Collective behavior– including grouping behavior (e.g., schooling, shoaling, flocking) and social structure (Couzin and Krause 2003; Sumpter 2006; Sih et al. 2009)– might also be expected to evolve in parallel because of its ecological importance, as tasks such as foraging, predator avoidance, and habitat selection depend on the coordinated activities of multiple conspecifics (Croft et al. 2009; King et al. 2018; Jolles et al. 2020). Indeed, in guppies, populations in high predation habitats form stronger, more cohesive schools (Seghers 1974; Magurran et al. 1992; Reznick and Travis 2019) and social networks with increased connectivity and strength (Kelley et al. 2011) relative to those in low predation habitats. Furthermore, Plath and Schlupp (2008) found parallel evolution of shoaling behavior in the Atlantic molly (*Poecilia mexicana*): populations in caves had a reduced tendency to shoal compared to surface dwelling counterparts, likely due to reduced predation risk and increased resource competition in cave environments.

Despite some evidence for parallelism of collective behavior, there are at least two reasons why collective behavior might not exhibit parallelism across populations. First, if certain social structures are necessary for group functioning regardless of ecological conditions, then collective behavior might be robust, and not vary across environments; we call this the ‘robust’ hypothesis. Support for this comes from studies in forked fungus beetles (*Bolitotherus cornutus*), where changes in the distribution of resources had minimal effect on social networks (Costello et al. 2022). Similarly, in feral goats (*Capra hircus*), social structure (social interaction rates, group composition, and group size) was not associated with habitat or resource quality (Stanley and Dunbar 2013). Second, collective behavior might evolve in an idiosyncratic, population-dependent way rather than in parallel; we call this the ‘population-dependent’ hypothesis. Under this hypothesis, collective behavior would still evolve, but evolutionary trajectories would not be predicted by habitat type (Oke et al. 2017; Bolnick et al. 2018). This could occur if there is fine grained environmental variation that is not accounted for by categorical habitat types. For instance, a study of lake- and stream- stickleback found that deviations from parallelism could be explained by incorporating quantitative data on fine-scale habitat features including depth, flow, and vegetation structure (Stuart et al. 2017). Collective behavior might tend to be influenced by the immediate, fine-grained social environment rather than canonical ecological drivers because collective behavior is the outcome of local interactions between individuals (Farine et al. 2015; Formica et al. 2021).

Here, we evaluate evidence for parallelism, robustness, and population-dependence in the evolution of collective behavior by comparing collective behavior across multiple populations of three-spined stickleback from Alaska. Stickleback are a classic model in the study of parallel evolution, with their propensity for rapid adaptation and repeated divergence of ecotype pairs (McKinnon and Rundle 2002). They are appropriate subjects for studying collective behavior because they form groups (shoals) while foraging, and these groups exhibit variation in cohesion, activity, social attraction, and social networks (Croft et al. 2005; Rystrom et al. 2019; MacGregor and Ioannou 2021). Previous work has also shown that collective behavior is repeatable (Jolles et al. 2018; Georgopoulou et al. 2022) and shoaling tendency has a genetic basis in stickleback (Wark et al. 2011; Greenwood et al. 2015) suggesting that collective behaviors have the potential to evolve in response to natural selection (Doucette et al. 2004; Olafsdottir et al. 2014; Neumann and Bell 2023).

Among the best studied examples of phenotypic divergence in stickleback are the benthic and limnetic ecotypes. The benthic ecotype occurs in small, shallow lakes, preying primarily on macroinvertebrates and crustaceans in the benthos, whereas the limnetic ecotype occurs in deeper lakes where zooplankton are the predominant food source (Bell and Foster 1994). Benthic-type fish tend to have deeper bodies and caudal peduncles, and shorter heads and snouts, compared to limnetic-type fish (Matthews et al. 2010; Willacker et al. 2010). We hypothesized that collective behavior diverges along thebenthic-limnetic axis because the limnetic ecotype tends to occur in open water, where there is minimal plant cover. Forming more social, cohesive groups could help counter any potential increases in predation risk due to this more open, exposed environment. Indeed, the limnetic ecotype exhibits an increased tendency to shoal (Vamosi and Schluter 2002; Kozak and Boughman 2012) and forages in groups more often than the benthic ecotype (Larson 1976).

To test this hypothesis, we studied stickleback from eight lakes in Alaska. These lakes can be roughly categorized as benthic- versus limnetic-type based on stickleback morphology (Hendry et al. 2024) and are derived from the same meta-population of marine stickleback, providing an opportunity to study parallel evolution (Bell and Foster 1994). Fish were reared in the lab to minimize environmental effects, as plasticity could influence phenotypic parallelism (Oke et al. 2016). We first measured a suite of morphological traits to verify that these populations exhibited the expected benthic-limnetic morphological differences and explored correlations between morphology and collective behavior. Next, we measured individual latency to emerge, providing an opportunity to assess evidence for parallel divergence of a non-social behavior relative to social behaviors (collective behavior) and because a previous study found that benthic stickleback are bolder (Keagy et al. 2023). Finally, to test whether there is divergence of collective behavior in line with benthic-limnetic divergence, we measured the collective behavior (activity, cohesion, social interaction rate, and clustering coefficient) of groups of stickleback from each of the eight populations. We repeated this twice per group to estimate the repeatability of collective behavior. We predicted that fish from limnetic populations would form more ‘social’ groups that are more cohesive and have greater social interaction rates and clustering coefficients compared to fish from benthic populations.

## Materials and methods

### Fish collection and husbandry

We captured adult three-spined stickleback from eight lakes in the Kenai Peninsula and Matanuska-Susitna Valley, Alaska, U.S.A. in June 2021, using minnow traps. We categorized four of these lakes as benthic and four as limnetic (Table 1), based on prior work by Hendry et al. (2024). This study quantified these lakes as benthic and limnetic based on various environmental characteristics (calcium, chlorophyll a, dissolved organic carbon, lake surface area, maximum depth, pH, specific conductance, total nitrogen, total phosphorus, dissolved oxygen) and morphological features of wild-caught fish sampled from the lakes. These populations likely diverged within the past 10,000 years, following glacial retreat and the subsequent colonization of stickleback into freshwater habitats (Rundle et al. 2000).

We artificially fertilized 2–4 clutches from each population and shipped them to the University of Illinois Urbana-Champaign. They were kept in tanks (32 L x 21 W x 19 H cm) with a bubbler until hatching. Fry were fed brine shrimp daily and transitioned to a diet of brine shrimp, blood worms, mysis shrimp, spirulina shrimp, and cyclops as they developed. At approximately one month of age, we relocated the juveniles to larger tanks (55 L × 33 W × 24 H cm) in single family groups of 8–15 individuals at 20 °C, on a 16 h light − 8 h dark photoperiod, in a recirculating system supplying 10% daily water changes. The sample sizes for Wik Lake and Tern Lake were low due to initial mortality of clutches after arrival to the lab (*n* = 16 fish, 2 groups, and 2 families per population).

It is important to note that these family groups differed in size, and prior work has shown that group size can have an impact on stickleback behavior (Frommen et al. 2009). We attempted to account for this by moving experimental fish into tanks of equal density for approximately two months prior to the start of the study but acknowledge that it is still possible that there was an effect of this early life experience on behavior.

### Elastomer marking and assignment of fish to experimental groups

In December 2021, we assigned 240 fish (~ 6 months old), 120 from benthic populations and 120 from limnetic populations, to 30 experimental groups, each containing eight fish from a single population in line with stickleback group sizes in the field (Ward et al. 2017). All groups were made up of multiple families, with 2–4 fish from each family depending on the number of families available (e.g., populations with four families received two per family). Because of differences in the number of fish available from each population, some populations had more groups than others (Table 1). Each group was kept in its own tank (55 L × 33 W × 24 H cm) (e.g., no other fish besides the experimental fish).

In January 2022, we marked each fish with a unique combination of two visual elastomer implant tags (0.5 cm long; colors mix of blue, yellow, purple, pink, and green) on the dorsal side of the body (Northwest Marine Technologies, U.S.A.). Elastomer tags fluoresced under ultraviolet light, allowing us to track the identity of all individuals. These tags were not visible to human observers. However, we know that visibility of elastomer colors can differ (Hohn and Petrie-Hanson 2013) and color may influence social interactions in other fish species (Frommen et al. 2015). Thus, it is possible that the tag identity could have had an effect on this study. After the marking period, we waited six weeks until proceeding to the next stage of the experiment. This waiting period also controlled for any effect of familiarity by making all fish in a group familiar to one another prior to any measurement of behavior (Atton et al. 2014).

**Table 1:** Overview of populations and experimental fish from each population

Population	Coordinates	Ecotype	Families	Experimental groups	Experimental fish
Finger Lake	61.60, -149.28	Benthic	4	7	56
Tern Lake	60.53, -149.55	Benthic	2	2	16
Walby Lake	61.62, -149.21	Benthic	4	3	24
Watson Lake	60.54, -150.46	Benthic	2	3	24
Long Lake	61.57, -149.76	Limnetic	3	4	32
Spirit Lake	60.59, -150.98	Limnetic	3	5	40
South Rolly Lake	61.66, -150.13	Limnetic	3	4	32
Wik Lake	60.72, -151.25	Limnetic	2	2	16

### Quantifying morphological variation

**Fig. 1:**
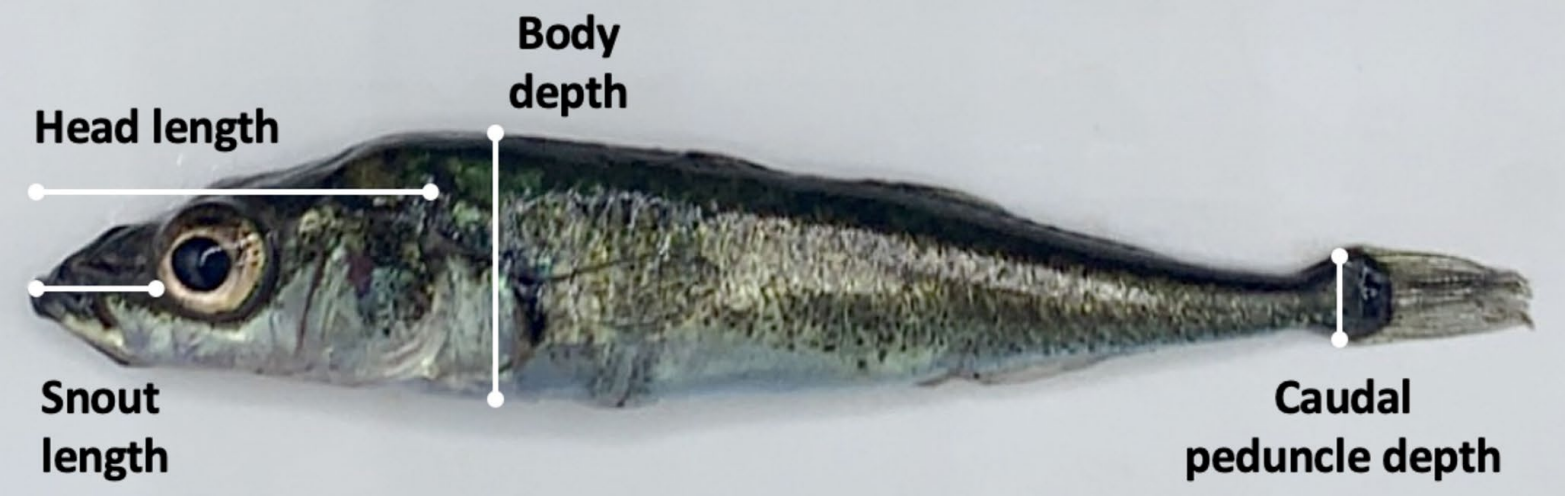
Morphological features measured

We measured the morphology of 159 of the fish in this experiment, from three populations per ecotype, excluding Wik and Tern Lake because individuals from these populations were being used for a different study following measures of collective behavior. We photographed individuals using an iPhone XS and used ImageJ to measure the following morphological features that are known to be associated with benthic-limnetic divergence: body depth, caudal peduncle depth, head length, and snout length (Fig. 1). We adjusted traits to correct for body size using the formula $\:{M}_{adj}={{M}_{o}(\underset{\_}{SL}/{SL}_{o})}^{b}$, where $\:{M}_{adj}$ is the adjusted trait, $\:{M}_{o}$ is the observed trait, $\:{SL}_{o}$ is the standard length, $\:\underset{\_}{SL}$ is the grand mean standard length, and $\:b$ is the slope of an ANCOVA of the form $\:M\:\sim\:SL+population$ (Reist 1986; Hendry and Taylor 2004; Haines et al. 2023).

### Latency to emerge assays

From February to March 2022, between 13:00 and 17:00 CST, we measured individual latencies to emerge. Each individual was moved in a cup of water from their home tank and gently poured into an opaque enclosed shelter (diameter = 11 cm, height = 9 cm) in a plexiglass tank (32 L x 21 W x 19 H cm). After five minutes of acclimation, we uncovered a 3 cm opening and recorded the fish using a JVC Everio GZ-HD40U camcorder. From the videos, we scored the time it took the fish to emerge. If a fish did not leave after ten minutes, they received a maximum score of 600 s. Here and in all subsequent assays, all fish were fed at the same time the day prior to behavioral measurements and were not fed the day of testing. Blinded methods were used during latency to emerge trials to minimize bias.

### Shoaling assays

Shoaling assays took place between 12:00 and 18:00 CST from March to April 2022. First, each of the eight individuals were placed into their own opaque plastic cup (diameter = 9 cm) inside a round, opaque plastic pool (diameter = 1.2 m, height = 8 cm). After 10 min, all cups were simultaneously removed and the group was filmed from above for 20 min using a JVC Everio GZ-HD40U camcorder. Groups were measured twice, with approximately two weeks between measurements. Fish were housed with their group in between trials. We chose this time to avoid the effect of aggression between males that can occur during the breeding season starting in May.

### Quantifying collective behaviors

We tracked the movement of all individuals in each video while retaining the identity of each fish using idTracker (Perez-Escudero et al. 2014). idTracker returns the x, y coordinates for the center of mass of each individual for each frame in the video. Thus, we had 480 total observations for each of the behaviors below: 240 individuals, each measured twice (except for the pairwise social interaction rates, which had 1680 observations, two for each possible pair of fish). We exclude any frames that did not identify all eight individuals. Missing frames accounted for *≤* 2% of the frames in a video. We computed the following behaviors:

1. Activity: distance swam.
2. Cohesion: average distance of individual from the centroid of the group.
3. Social interaction rate: time each pair of fish spent within 1 body length of one another.
4. Network metrics
  We further explored collective behaviors by constructing weighted social networks with the ‘igraph’ package (Csárdi and Nepusz 2006; Csárdi et al. 2025). Nodes were individuals and edges were the social interaction rate between individuals. We computed two network metrics:
  - Strength: sum of all edges associated with a node, providing a measure of social interaction (similar to degree, but for a weighted network).
  - Clustering: local clustering coefficients for weighted networks were computed using the ‘DirectedClustering’ package (Clemente and Grassi 2018). Higher values of clustering suggest that the individuals that one individual is interacting with often are also interacting with one another often (e.g., their “friends” are also “friends” with one another).

### Data analysis

All data analysis was conducted in R version 4.4.0 (R Core Team 2024). For both collective behavior and morphology, we performed principal component analysis (PCA) because initial inspection of data identified many correlations (Online Resource 1). We took the mean of each fish’s behavior across the two trials. Individual latency to emerge was not included because it was measured in a separate assay and initial inspection did not find strong correlations (Online Resource 1). For both PCAs, we analyzed the top two principal components (PCs), which accounted for 79% of the variance in the morphology PCA (hereafter ‘PCA-M’) (Table 2) and 87.3% of the variance in the collective behavior PCA (hereafter ‘PCA-CB’) (Table 3).

**Table 2:** Loadings for PC1 and PC2 for morphology

Morphological trait	Loading PC1 (0.51 variance explained)	Loading PC2 (0.28 variance explained)
Body depth	-0.384	0.684
Caudal peduncle depth	-0.542	0.372
Snout length	-0.551	-0.405
Head length	-0.504	-0.478

**Table 3:** Loadings for PC1 and PC2 for collective behavior

Collective behavior	Loading PC1 (0.609 variance explained)	Loading PC2 (0.264 variance explained)
Activity	-0.081	-0.951
Strength	0.603	-0.141
Cohesion	-0.565	-0.199
Clustering	0.557	-0.188

To compare the morphological and behavioral traits across populations and ecotypes, we took a linear mixed modeling approach. We could not include both population and ecotype as fixed effects in the same model because population is nested within ecotype, leading to perfect multicollinearity between these variables when they are included as fixed effects. Thus, we ran separate models. First, to assess the effect of ecotype, we fit linear mixed models (or generalized linear mixed models) with PCA-M score or PCA-CB score as the response, ecotype as a fixed effect, and population and family nested within population as random effects. For collective behavior models, we also had a random effect of group. The scores for the top two principal components for morphology and collective behavior, respectively, were approximately normally distributed, allowing us to run linear mixed models. For latency to emerge, we ran a generalized linear mixed model with a quasi-Poisson family. All linear models were run using the ‘lme4’ package (Bates et al. 2015), except for the latency to emerge model, which used the ‘glmmTMB’ package (Brooks et al. 2017). We computed the significance of the fixed effect of population using the Anova function in the ‘car’ package with Type III sum of squares (Fox and Weisberg 2019). We interpreted a significant effect of ecotype in this model as support for the parallel hypothesis.

To assess the effect of population, we ran another set of models with the same structure as above, except not including ecotype and with population as a fixed effect. We again used the Anova function to assess significance, and then made post-hoc comparisons (when necessary) at the pairwise level by comparing the estimated marginal means using the ‘emmeans’ package (Lenth 2025). We interpret a significant effect of population in this model as support for the population-dependent hypothesis, and no effect of ecotype or population across these and the prior models as support for the robust hypothesis.

To measure consistency of collective behavior across the two trials, we computed the adjusted repeatability for each behavior, which accounts for variation due to factors (e.g., population) through mixed modeling (Nakagawa and Schielzeth 2010). We ran linear mixed models with the behavior as the response, population as a fixed effect and fish ID as a random effect, using the ‘rptR’ package (Stoffel et al. 2017). We used a log transformation for activity and a square root transformation for cohesion and strength. Clustering was approximately normally distributed. We calculated 95% confidence intervals and p-values with parametric bootstrapping (*n* = 10,000 iterations) and p-values from permutation tests (*n* = 10,000 iterations). We also looked at the repeatability of pairwise interaction rates. While strength represents the sum of all social interactions associated with an individual, pairwise interaction rate gives the amount of time each pair of fish interacted with one another. This allowed us to assess if individuals form consistent social relationships. We did not include pairwise interaction rate in other analyses because it is strongly correlated with strength.

To determine if there were correlations between behavior and morphology, we computed Spearman’s rank correlation coefficients between PCA-M scores, individual latency to emerge, and PCA-CB scores.

## Results

### Morphology

PC1-M had high negative loadings for all morphological variables. Thus, fish with either significantly higher or significantly lower scores on PC1-M were generally more ‘extreme’ in their morphology. Meanwhile, PC2-M had a high positive loading for body depth. Thus, fish with higher scores on PC1 had deeper bodies.

There was no significant effect of ecotype on PC1-M score (Chi-square = 3.24, df = 1, *n* = 159, *p* = 0.072), but there was a significant effect of population (Chi-square = 23.81, df = 1, *n* = 159, *p* < 0.01). Specifically, fish from Long Lake had significantly higher scores on PC1-M (Online Resource 1), suggesting that they had shorter heads and snouts and shallower bodies and caudal peduncles.

**Fig. 2:**
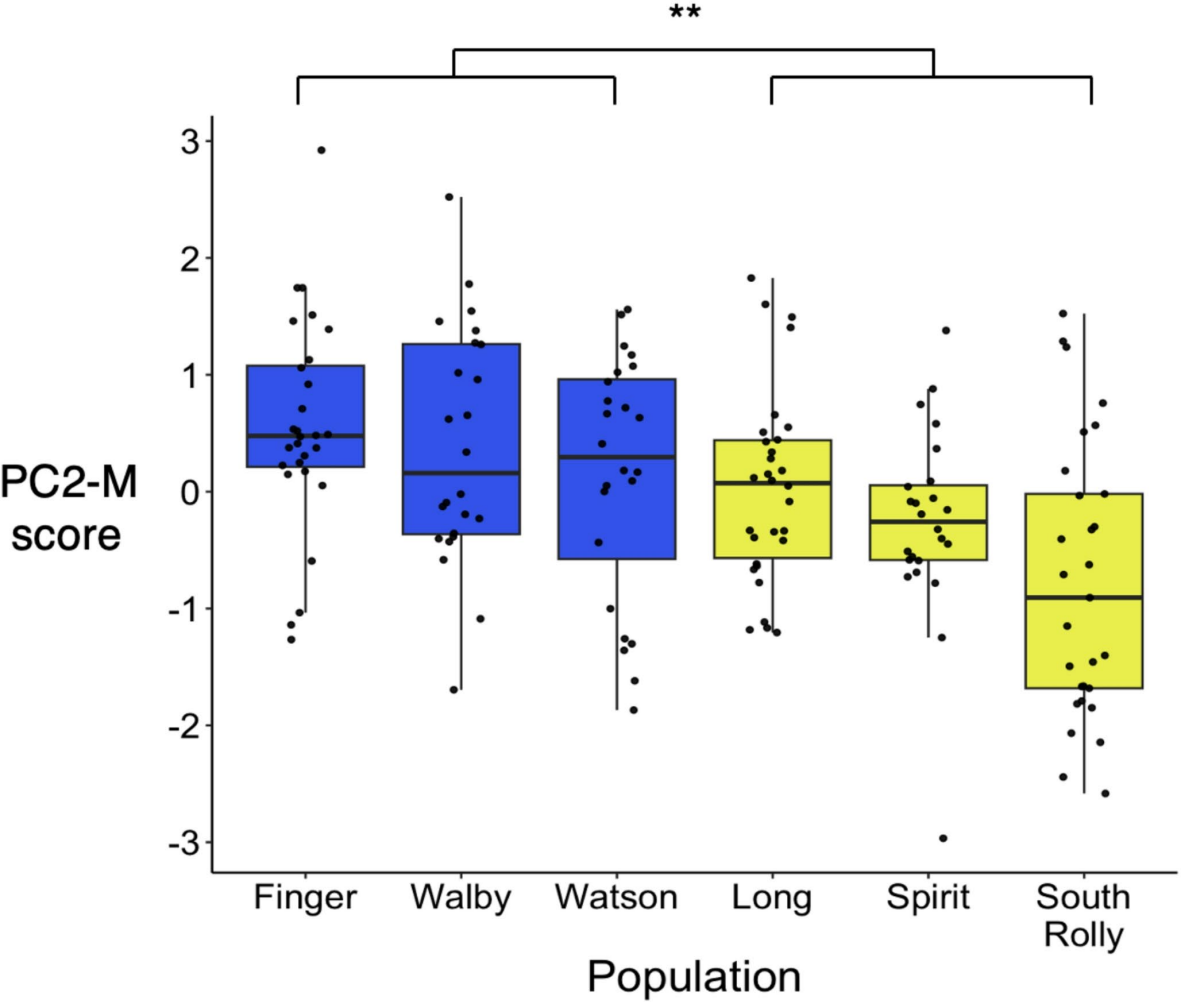
Fish from benthic populations had deeper bodies than fish from limnetic populations. High scores on PC2-M correspond to deeper bodies. Blue = benthic population, yellow = limnetic population (*n* = 159). Here and in all following Figs, data points represent individual fish; boxes represent the interquartile range (IQR), the central line within boxes represent the median, and whiskers represent 1.5 * IQR; and asterisks indicate significant differences (one asterisk: *p* < 0.05, two asterisks: *p* < 0.01)

There was a significant effect of ecotype on PC2-M (Chi-square = 6.77, df = 1, *n* = 159, *p* < 0.01, Fig. 2), suggesting that fish from benthic populations had significantly deeper bodies than fish from limnetic populations. There was also a significant effect of population (Chi-square = 31.99, df = 5, *n* = 159, *p* < 0.0001), with post-hoc comparisons supporting the finding that fish from benthic populations had deeper bodies, in line with our predictions (Online Resource 1).

### Individual latency to emerge

**Fig. 3:**
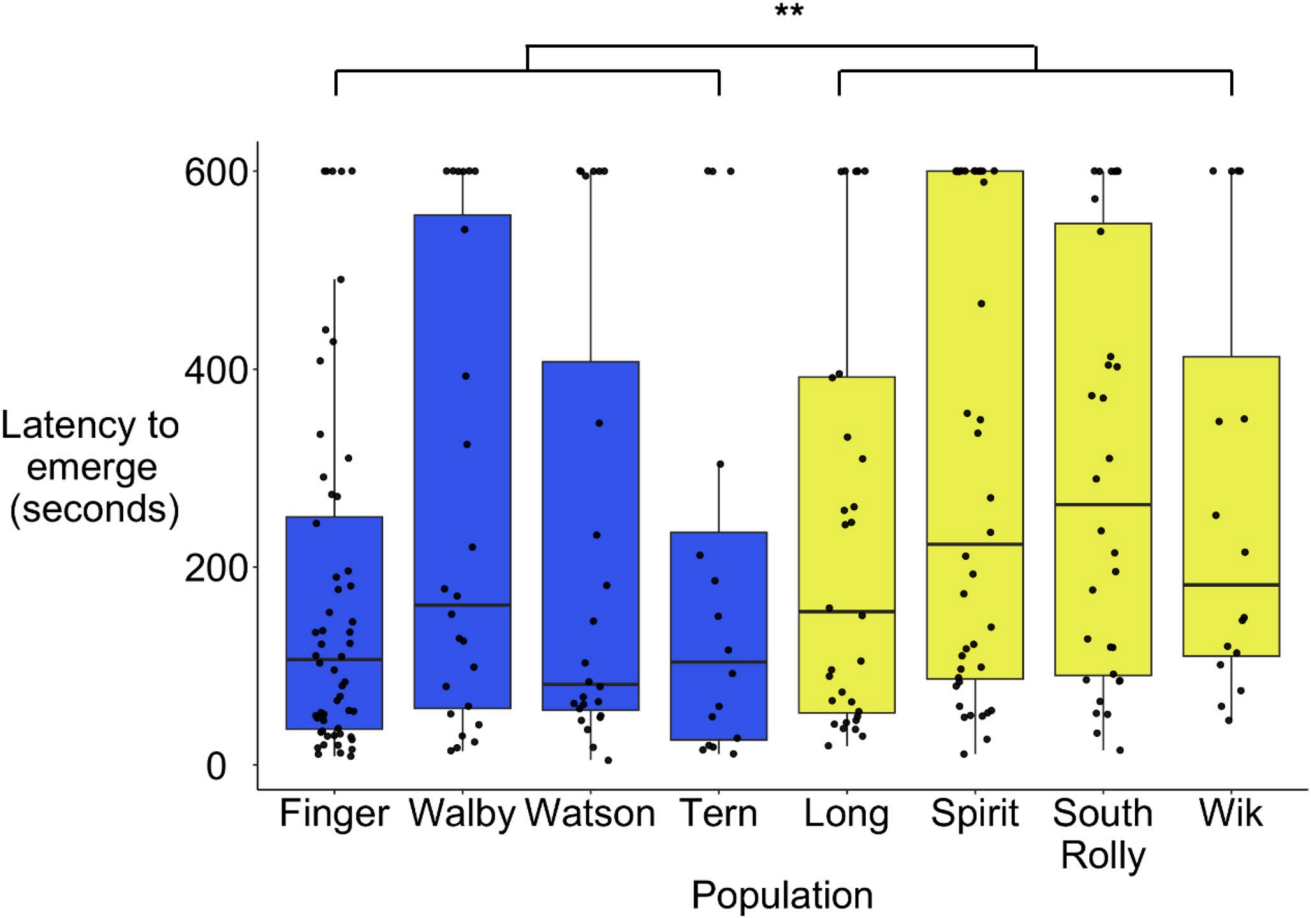
Fish from benthic populations fish emerged faster from a shelter than fish from limnetic populations. Blue = benthic population, yellow = limnetic population (*n* = 240). Boxes represent the interquartile range (IQR), the central line within boxes represent the median, and whiskers represent 1.5 * IQR; and asterisks indicate significant differences (one asterisk: p < 0.05, two asterisks: p < 0.01)

Consistent with prior studies that suggested benthic stickleback are bolder than limnetic stickleback (Keagy et al. 2018), fish from benthic populations emerged from a shelter faster than fish from limnetic populations (Chi-square = 8.24, df = 1, *n* = 240, *p* < 0.01; Fig. 3). In line with this, there was also significant population-level variation (Chi-square = 26.65, df = 7, *n* = 240, *p* < 0.001), with Finger as the boldest population (Online Resource 1).

### Repeatability of collective behavior

We computed the adjusted repeatability for all collective behaviors, which accounted for variation among populations. All of the repeatabilities were significantly different from zero (Table 4).

**Table 4:** Collective behavior was repeatable. Repeatability measures, adjusted for population, are reported, with 95% confidence intervals (*n* = 240)

	Activity	Cohesion	Strength	Pairwise interaction rate	Clustering
Adjusted repeatability	**0.46** **(0.35–0.56**, ***p*** < **0.001)**	**0.21** **(0.08–0.33**, ***p*** < **0.01)**	**0.63** **(0.55–0.70**, ***p*** < **0.00001)**	**0.49** **(0.44–0.54**, ***p*** < **0.001)**	**0.18** **(0.05–0.30**, ***p*** < **0.01)**

### Collective behavior

PC1-CB had high loadings for strength, cohesion, and clustering. Lower values of cohesion indicate more cohesive groups (i.e., shorter distances to centroid of the group). Thus, fish with relatively high scores on PC1-CB were more cohesive, more social (higher strength), and in more tightly connected social circles (fish that the focal fish interacted with more also interacted with one another more). PC2-CB had a high loading for activity, such that fish with higher scores on PC2-CB were less active.

There was no significant effect of ecotype on PC1-CB scores (Chi-square = 0.43, df = 1, *n* = 240, *p* = 0.51, Fig. 4a). However, there was a significant effect of population (Chi-square = 23.81, df = 7, *n* = 240, *p* < 0.01). Post-hoc analysis found that fish from Long Lake had significantly higher scores than Finger, Watson, and Spirit. Additionally, fish from Watson Lake had significantly lower scores than Walby (Online Resource 1). Thus, fish from Long were the most cohesive, social, and in the most tightly connected social circles of all populations, while fish from Watson were on the other end of the spectrum.

**Fig. 4a:**
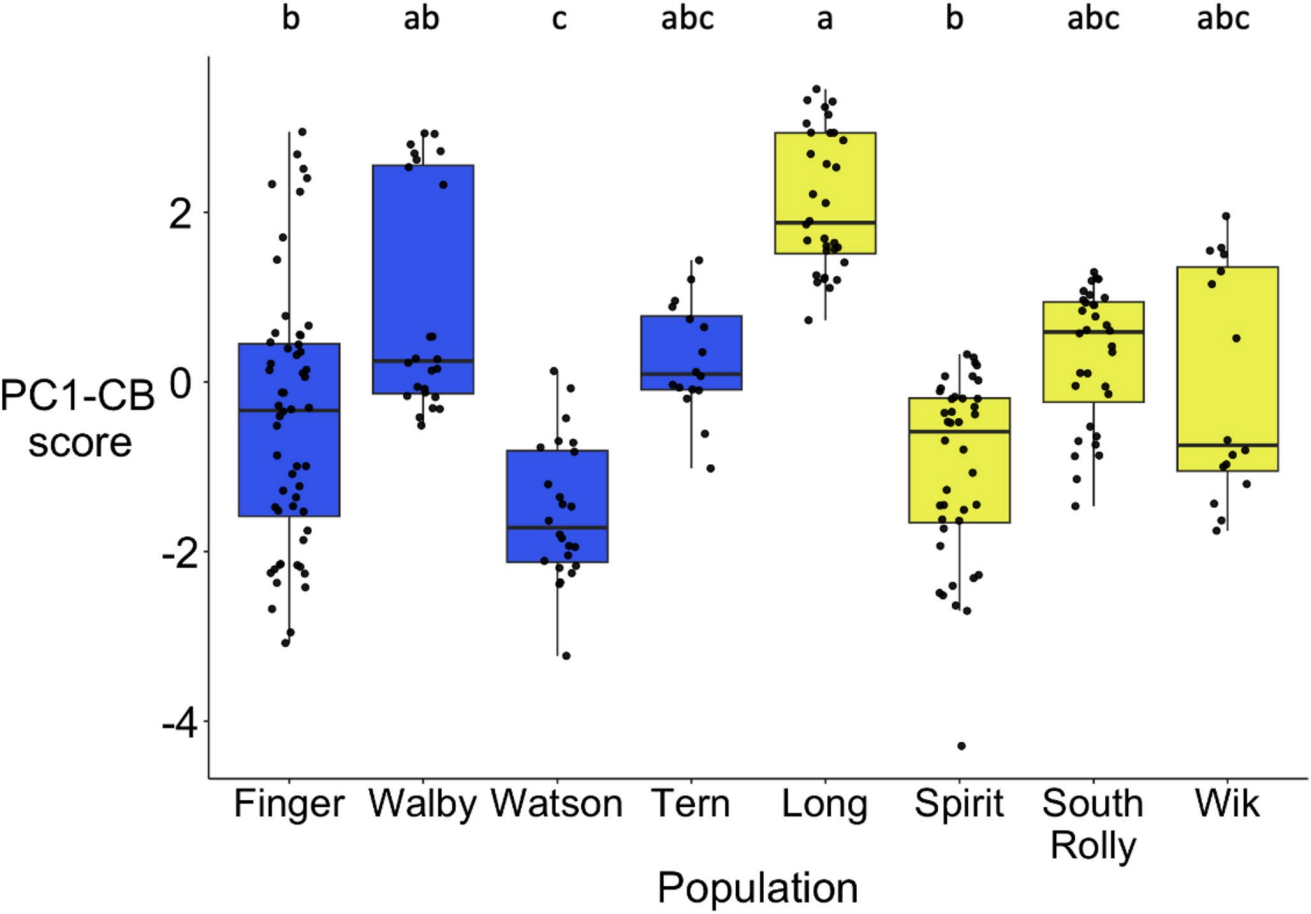
There were significant differences in collective behavior among populations, but no evidence for consistent differences between benthic and limnetic populations. High scores on PC1-CB correspond to more social groups (higher values for strength and clustering and lower values for cohesion). Blue = benthic population, yellow = limnetic population (*n* = 240). Letters indicate significant differences between populations.Boxes represent the interquartile range (IQR), the central line within boxes represent the median, and whiskers represent 1.5 * IQR; and asterisks indicate significant differences (one asterisk: p < 0.05, two asterisks: p < 0.01)

There was no significant effect of ecotype on PC2-CB scores (activity) (Chi-square = 2.13, df = 1, *p* = 0.14, Fig. 4b), but there was a significant effect of population (Chi-square = 52.87, df = 7, *p* < 0.00001). Post-hoc analysis found that Finger had significantly lower scores on PC2-CB than all other populations besides Long, corresponding to higher activity (Online Resource 1).

**Fig. 4b:**
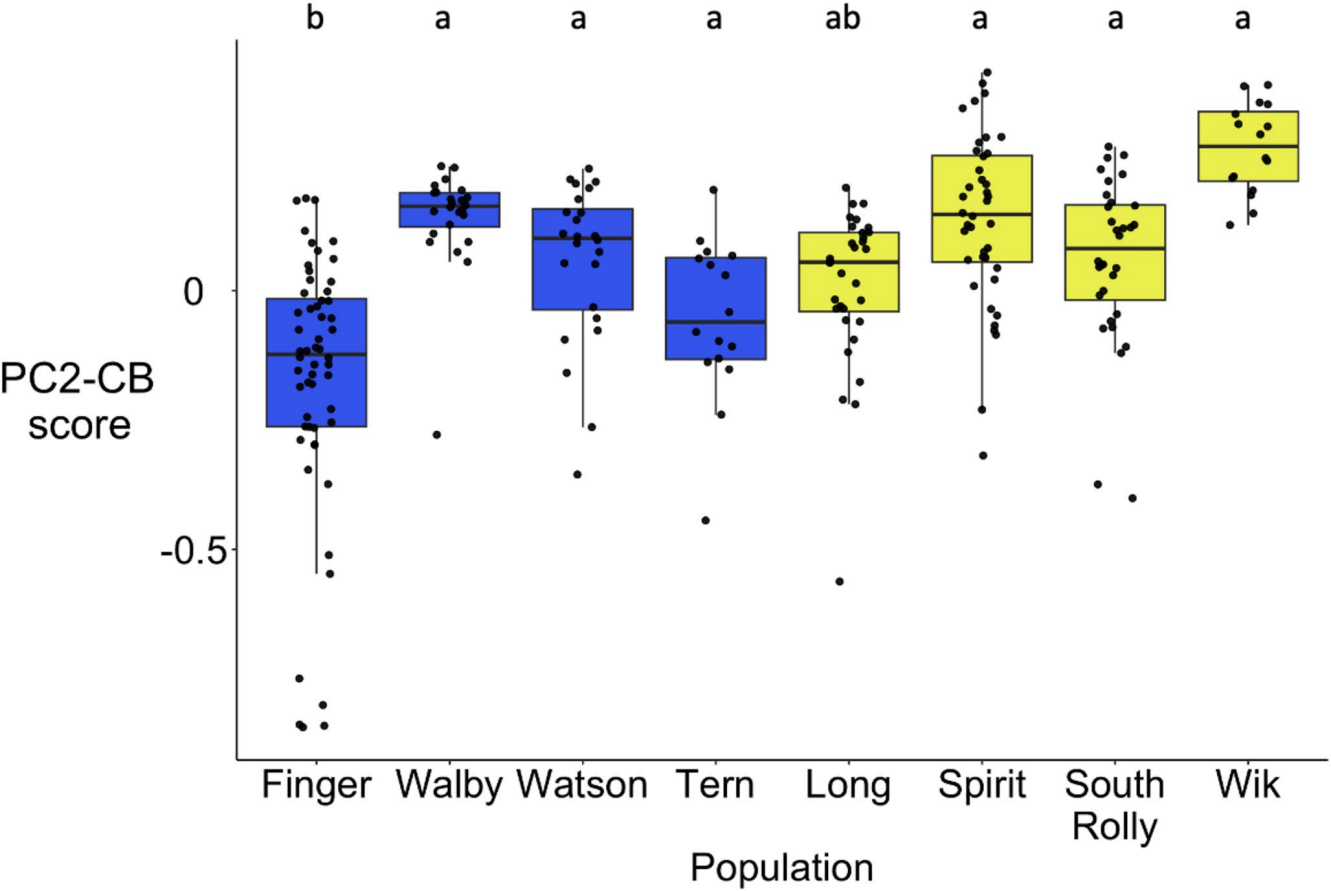
There were significant population-level differences in activity, but no evidence for consistent differences in activity between benthic and limnetic populations. High scores on PC2-CB correspond to lower activity because activity had a strong negative loading for PC2-CB. Data points are symmetric log transformed for visualization. Blue = benthic population, yellow = limnetic population (*n* = 240). Letters indicate significant differences between populations. Data points represent individual fish; Boxes represent the interquartile range (IQR), the central line within boxes represent the median, and whiskers represent 1.5 * IQR

### Relationship between behavior and morphology

There were no consistent relationships between morphology and collective behavior, which suggests that these traits are evolving independently of one another (Online Resource 1). Individual latency to emerge was also not correlated with any of the collective behaviors (Online Resource 1). There was a significant negative correlation between latency to emerge and PC2-M score, suggesting that bolder fish (shorter latency to emerge) tended to have deeper bodies (Rho = -0.22 (95% CI: -0.37–0.063), *n* = 159, *p* < 0.01).

## Discussion

We asked whether collective behavior consistently varies across replicate benthic and limnetic populations of stickleback in Alaska. Previous studies suggest that an entire suite of morphological (body shape, feeding morphology, gill rakers, lateral lines, brain size) and behavioral (latency to emerge, reproductive behavior) traits have evolved in parallel with benthic-limnetic divergence in sticklebacks (Foster et al. 2008; Wark and Peichel 2010; Willacker et al. 2010; Keagy et al. 2018, 2023). Given its ecological importance, we hypothesized that collective behavior would also exhibit evidence for parallel evolution. Our results show that collective behavior can evolve — it was repeatable, and there was extensive population-level variation in a common lab environment. However, this variation was not consistently aligned along the benthic-limnetic axis.

Fish from benthic populations had significantly deeper bodies (greater PC2-M scores), even when reared in a common lab environment. This is consistent with prior studies on benthic and limnetic populations, giving us confidence that our study captured the axis of divergence we aimed to compare. However, we did not detect significant differences in principal components associated with caudal peduncle depth, head length, or snout length, other morphological traits that have been shown to differ between these ecotypes. The failure to detect parallelism of these traits might reflect plasticity, as has been found in prior studies on lab-reared stickleback (Day et al. 1994; Svänback and Schluter 2012).

One explanation for the finding that there was population-level, but not ecotypic, variation in collective behavior is that the evolution of collective behavior is driven by other environmental factors that are not aligned with benthic-limnetic divergence, such as predation risk. Limnetic populations might face greater predation risk because they spend more time foraging in open water, leading to more social groups (Larson 1976; Kozak and Boughman 2012). However, if the density of predators is low in a limnetic population, it could reduce predation risk even in open water, leading to less social groups (less cohesive, lower social interaction rates, lower clustering; i.e., lower values in Fig. 4b). Work in other stickleback populations has found that individual antipredator behavior evolves in parallel across populations that face similar predation regimes (Wund et al. 2015). Future research could investigate if collective responses to predators follow a similar pattern, as these populations can encounter a wide diversity of predators including pike, trout, salmon, and aquatic birds. From this study, fish from Long, Watson, and Finger Lake had the most extreme measures of collective behavior, with more social behavior for Long, less social behavior for Watson (Fig. 4a), and high activity for Finger (Fig. 4b), making these populations strong candidates for further research.

Future studies could also explore the functional aspects of these population differences in collective behavior. For instance, prior work in stickleback has found that social network structure influences the discovery of foraging patches (Atton et al. 2014), and that quorum decision-making drives collective responses to predation (Ward et al. 2008). It is possible that the observed differences among populations in our study are associated with differences in decision-making strategies across foraging and/or predation contexts. This would suggest that adaptive evolution is occurring in these populations independently of benthic-limnetic habitat differences.

While we have thus far focused on the consequences of our between-population comparisons, we also found considerable within-population variation in morphology and behavior. Prior work suggests that allopatric populations of benthic and limnetic stickleback, like the ones in this study, tend to show greater levels of morphological variation than sympatric or marine populations (Willacker et al. 2010; Svanbäck and Schluter 2012). It could be that there are microhabitat differences within lakes driving this within-population variation (Maciejewski et al. 2020). While there is less work exploring within-population variation of behavior in these ecotypes, shoaling behavior is known to be plastic in response to the predation environment (Kozak and Boughman 2012). Thus, the potential effects of predation discussed in the previous paragraph from a between-population perspective could also contribute to within-population variation, if stickleback experience different predator pressure within the same lake.

Stickleback from benthic populations were bolder, consistent with Keagy et al. (2023), and bolder fish had deeper bodies, typical of the benthic ecotype. Benthic stickleback tend to forage in more sheltered habitats, which might make them more willing to take risks (Hart and Gill 1994). A similar pattern has been detected in guppies, where there was evidence for parallelism of boldness but not collective behavior (shoaling) across populations from high- and low-predation environments (Jacquin et al. 2016). Notably, Jacquin et al. still found evidence for evolution of shoaling, just in a non-parallel manner. This evidence for parallelism of boldness, both in Jacquin et al. and our study, suggests that behavior is capable of evolving in these systems, and the failure to detect parallel evolution of collective behavior is not because behavioral traits are more labile in general. Instead, it could be that there is something different about collective behavior relative to other types of behavior that might make it less likely to exhibit parallelism. One possibility is that the effects of the social environment on collective behavior exceeded the effects of benthic- versus limnetic-type habitats. For example, the phenotypic composition of individuals in a group might be more important than benthic/limnetic phenotype in shaping the behavior of stickleback groups (Jolles et al. 2015; Kim et al. 2022). In zebra finches (*Taeniopygia guttata*), differences in social connectivity emerged even when accounting for ecological differences (Ogino et al. 2023), and in European shore crabs (*Carcinus maenas*), individual connectivity in social groups (‘betweenness’) was consistent even when the distribution of resources changed (Tanner and Jackson 2012). Thus, it is possible that the social environment influences collective behavior more than other ecological conditions.

We also found that repeatable differences in collective behavior can emerge in a common lab environment, suggesting that these are enduring group-level traits. Previous work has suggested that schooling and shoaling behavior in sticklebacks has a genetic basis (Wark et al. 2011; Greenwood et al. 2015; Gaffney and Webster 2018). Furthermore, the repeatability of pairwise interaction rates suggests that, despite not forming long-term social groups, stickleback may still recognize and form social relationships with other individuals, as has been found in mark-recapture studies in the field (Ward et al. 2002). This adds to growing evidence that stickleback are a strong model for studying the evolution of social networks (Webster et al. 2013; Kleinhappel et al. 2014), as has been done in other model organisms like mice and bees (So et al. 2015; Traniello et al. 2022).

While we did not detect systematic differences in collective behavior among replicate benthic and limnetic populations, previous studies have found that limnetic stickleback have a higher tendency to shoal (Vamosi 2002; Kozak and Boughman 2012). It could be that while general shoaling tendency might be greater in limnetics, other environmental features like predation or competition could influence behavior once the group is formed, resulting in the population-dependent patterns of collective behavior we observed. These studies looked at sympatric benthic and limnetic stickleback from a single population (Paxton Lake), so it is also possible that this pattern is not consistent when looking at allopatric populations like the ones in our study. Another caveat is that we used only morphology to categorize populations. These populations might not necessarily be divergent in other benthic-limnetic traits; for instance, feeding morphology or immunology (Schluter and McPhail 1992; Stutz et al. 2014). Thus, any conclusions about the relationship between collective behavior and benthic-limnetic traits beyond linear morphometrics should be speculative until further data are collected. Finally, it could be that there is plasticity in collective behavior, and differences might emerge in wild-caught fish. Future work in this area is needed as all current work quantifying shoaling in these ecotypes has been done on lab-reared fish.

These findings indicate that collective behavior evolves, but not necessarily in a way that is predicted by benthic versus limnetic habitat type. Meanwhile, for morphology and latency to emerge, there was support for consistent differences across replicate benthic and limnetic populations. Thus, by examining multiple traits, we demonstrate that environmental differences can shape phenotypic evolution in parallel and non-parallel ways. By considering the role of other environmental characteristics beyond the benthic-limnetic axis, including predation, competition, or the social environment, on behavior and morphology, future research can begin to uncover how selection might be acting at different scales, from the level of interactions between individuals up to environmental features.

## Electronic supplementary material

Below is the link to the electronic supplementary material.

Supplementary Material 1

## Data Availability

Data available from the Dryad Digital Repository: 10.5061/dryad.2280gb620 (Neumann et al. 2025).
