# Supplementary material for: Collective behavior diverges independently of the benthic-limnetic axis in stickleback
Source: Behav Ecol Sociobiol. 2025 May 8;79(5):56. doi: 10.1007/s00265-025-03599-z (PMC12058956; doi:10.1007/s00265-025-03599-z)
Supplement: Supplementary file 1 — Supplementary Material 1 [file 265_2025_3599_MOESM1_ESM.docx]

**Article:** Collective behavior evolves independently of benthic-limnetic divergence in stickleback. **Authors:** Kevin M. Neumann, Lucas Eckert, Damaris Miranda, Andrew Kemp, and Alison Bell

**Journal:** *Behavioral Ecology and Sociobiology*

**Corresponding author:**

Kevin M. Neumann

kevinn4@illinois.edu

University of Illinois Urbana-Champaign

Program in Ecology, Evolution, and Conservation Biology

**Supplemental Table S1a.** Correlations among morphological traits, after adjustment for body size. Right side of table displays Spearman’s correlations and left side of table displays p-values.

|  | **Body depth** | **Caudal peduncle depth** | **Head length** | **Snout length** |
| --- | --- | --- | --- | --- |
| **Body depth** | -- | 0.59 | 0.42 | 0.39 |
| **Caudal peduncle depth** | **<0.0001** | -- | 0.46 | 0.55 |
| **Head length** | **<0.0001** | **<0.0001** | -- | 0.76 |
| **Snout length** | **<0.0001** | **<0.0001** | **<0.0001** | -- |

**Supplemental Table S1b.** Correlations among collective behaviors. Right side of table displays Spearman’s correlations and left side of table displays p-values.

|  | **Activity** | **Cohesion** | **Clustering** | **Strength** |
| --- | --- | --- | --- | --- |
| **Activity** | -- | 0.24 | 0.02 | 0.01 |
| **Cohesion** | **<0.001** | -- | -0.59 | -0.77 |
| **Clustering** | 0.76 | **<0.0001** | -- | 0.78 |
| **Strength** | 0.81 | **<0.0001** | **<0.0001** | -- |

**Supplemental Table 2a.** Output of linear mixed model for type / PC1-M scores.

| **Effect** | **Chi-sq** | **df** | **p-value** |
| --- | --- | --- | --- |
| Intercept | 1.99 | 1 | 0.158 |
| Type | 3.24 | 1 | 0.072 |

**Supplemental Table 2b.** Output of linear mixed model for population / PC1-M scores.

| **Effect** | **Chi-sq** | **df** | **p-value** |
| --- | --- | --- | --- |
| Intercept | 0.14 | 1 | 0.70 |
| Population | 23.81 | 5 | 5.25e-08 |

**Supplemental Table 2c.** Output of linear mixed model for type / PC2-M scores.

| **Effect** | **Chi-sq** | **df** | **p-value** |
| --- | --- | --- | --- |
| Intercept | 3.55 | 1 | 0.059 |
| Type | 6.77 | 1 | 0.0092 |

**Supplemental Table 2d.** Output of linear mixed model for population / PC2-M scores.

| **Effect** | **Chi-sq** | **df** | **p-value** |
| --- | --- | --- | --- |
| Intercept | 8.48 | 1 | 0.0036 |
| Population | 31.99 | 5 | 5.975e-06 |

**Supplemental Table 2e.** Output of linear mixed model for type / boldness.

| **Effect** | **Chi-sq** | **df** | **p-value** |
| --- | --- | --- | --- |
| Intercept | 4298.12 | 1 | 2.2e-16 |
| Type | 8.24 | 1 | 0.0041 |

**Supplemental Table 2f.** Output of linear mixed model for population / boldness.

| **Effect** | **Chi-sq** | **df** | **p-value** |
| --- | --- | --- | --- |
| Intercept | 2346.72 | 1 | 2.2e-16 |
| Population | 26.65 | 7 | 0.00038 |

**Supplemental Table 2g.** Output of linear mixed model for type / PC1-CB scores.

| **Effect** | **Chi-sq** | **df** | **p-value** |
| --- | --- | --- | --- |
| Intercept | 0.14 | 1 | 0.71 |
| Type | 0.43 | 1 | 0.51 |

**Supplemental Table 2h.** Output of linear mixed model for population / PC1-CB scores.

| **Effect** | **Chi-sq** | **df** | **p-value** |
| --- | --- | --- | --- |
| Intercept | 0.94 | 1 | 0.33 |
| Population | 23.81 | 7 | 0.0012 |

**Supplemental Table 2i.** Output of linear mixed model for type / PC2-CB scores.

| **Effect** | **Chi-sq** | **df** | **p-value** |
| --- | --- | --- | --- |
| Intercept | 0.35 | 1 | 0.55 |
| Type | 2.13 | 1 | 0.14 |

**Supplemental Table 2j.** Output of linear mixed model for population / PC2-CB scores.

| **Effect** | **Chi-sq** | **df** | **p-value** |
| --- | --- | --- | --- |
| Intercept | 31.33 | 1 | 2.178e-08 |
| Population | 52.87 | 7 | 3.933e-09 |

**Supplemental Table S3a.** Comparisons of estimated marginal means for PC1-M across populations.

| **Comparison** | **Estimate** | **Standard error** | **t-ratio** | **p-value** |
| --- | --- | --- | --- | --- |
| FG - LG | -0.77392087 | 0.3436418 | -2.2521149 | 0.322500525 |
| FG - SL | 0.30447731 | 0.3653057 | 0.8334863 | 0.953185601 |
| FG - SR | -0.96474043 | 0.3465099 | -2.7841645 | 0.16889411 |
| FG - WB | 0.9864566 | 0.3630409 | 2.7172053 | 0.163084265 |
| FG - WT | 0.22396112 | 0.3666751 | 0.610789 | 0.985545497 |
| LG - SL | 1.07839818 | 0.3565934 | 3.0241671 | 0.131007948 |
| LG - SR | -0.19081956 | 0.3373125 | -0.5657056 | 0.989672978 |
| LG - WB | 1.76037747 | 0.3542729 | 4.9689867 | 0.014468034 |
| LG - WT | 0.99788199 | 0.3579962 | 2.7874095 | 0.226127912 |
| SL - SR | -1.26921774 | 0.3593581 | -3.5319022 | 0.068927141 |
| SL - WB | 0.68197929 | 0.3753237 | 1.8170431 | 0.502710142 |
| SL - WT | -0.08051619 | 0.3788401 | -0.2125334 | 0.999897567 |
| SR - WB | 1.95119703 | 0.3570556 | 5.4646872 | 0.008060764 |
| SR - WT | 1.18870155 | 0.3607501 | 3.2950829 | 0.139638884 |
| WB - WT | -0.76249549 | 0.3766567 | -2.024378 | 0.431481713 |

**Supplemental Table S3b.** Comparisons of estimated marginal means for PC2-M across populations.

| **Comparison** | **Estimate** | **Standard error** | **t-ratio** | **p-value** |
| --- | --- | --- | --- | --- |
| FG - LG | 0.5168124 | 0.2645158 | 1.9538057 | 0.44470592 |
| FG - SL | 0.8102895 | 0.2810018 | 2.883574 | 0.1303759 |
| FG - SR | 1.3205697 | 0.2666659 | 4.9521503 | 0.01202013 |
| FG - WB | 0.1622769 | 0.2782432 | 0.5832198 | 0.98977191 |
| FG - WT | 0.403014 | 0.2827526 | 1.4253236 | 0.71586799 |
| LG - SL | 0.293477 | 0.2743855 | 1.0695793 | 0.87890048 |
| LG - SR | 0.8037572 | 0.2596846 | 3.0951288 | 0.14055076 |
| LG - WB | -0.3545355 | 0.2715596 | -1.3055531 | 0.77497303 |
| LG - WT | -0.1137985 | 0.2761782 | -0.4120473 | 0.99733319 |
| SL - SR | 0.5102802 | 0.2764588 | 1.8457728 | 0.49627438 |
| SL - WB | -0.6480125 | 0.2876422 | -2.2528423 | 0.30531143 |
| SL - WT | -0.4072755 | 0.2920065 | -1.3947479 | 0.73105797 |
| SR - WB | -1.1582927 | 0.2736544 | -4.2326843 | 0.02966993 |
| SR - WT | -0.9175557 | 0.2782382 | -3.297734 | 0.13539997 |
| WB - WT | 0.240737 | 0.2893528 | 0.8319843 | 0.94949902 |

**Supplemental Table S3c.** Comparisons of estimated marginal means for boldness across populations.

| **Comparison** | **Estimate** | **Standard error** | **t-ratio** | **p-value** |
| --- | --- | --- | --- | --- |
| FG - LG | -0.419560551 | 0.1599246 | -2.62348937 | 0.147360438 |
| FG - SL | -0.630801555 | 0.1598161 | -3.94704667 | 0.002022158 |
| FG - SR | -0.627610014 | 0.1598501 | -3.92624131 | 0.002199008 |
| FG - TL | -0.105492754 | 0.1818178 | -0.58021126 | 0.99910629 |
| FG - WB | -0.440450542 | 0.1483596 | -2.96880344 | 0.059822496 |
| FG - WK | -0.532894368 | 0.181409 | -2.93753026 | 0.06535212 |
| FG - WT | -0.3454468 | 0.1812937 | -1.9054544 | 0.547066996 |
| LG - SL | -0.211241004 | 0.1707101 | -1.23742514 | 0.920842127 |
| LG - SR | -0.208049463 | 0.1707419 | -1.21850267 | 0.926730889 |
| LG - TL | 0.314067797 | 0.1914653 | 1.64033835 | 0.725679551 |
| LG - WB | -0.020889991 | 0.1600371 | -0.13053217 | 0.999999967 |
| LG - WK | -0.113333817 | 0.1910757 | -0.59313566 | 0.99896848 |
| LG - WT | 0.074113751 | 0.1909662 | 0.38809877 | 0.999938814 |
| SL - SR | 0.003191541 | 0.1706404 | 0.01870332 | 1 |
| SL - TL | 0.525308801 | 0.1913746 | 2.74492449 | 0.109381418 |
| SL - WB | 0.190351013 | 0.1599286 | 1.19022491 | 0.934976048 |
| SL - WK | 0.097907186 | 0.190985 | 0.51264337 | 0.999603974 |
| SL - WT | 0.285354755 | 0.1908754 | 1.49497893 | 0.810494753 |
| SR - TL | 0.52211726 | 0.191403 | 2.72784273 | 0.114209162 |
| SR - WB | 0.187159472 | 0.1599626 | 1.17002017 | 0.94046719 |
| SR - WK | 0.094715645 | 0.1910134 | 0.49585868 | 0.999682464 |
| SR - WT | 0.282163213 | 0.1909038 | 1.47803836 | 0.819428049 |
| TL - WB | -0.334957788 | 0.1819163 | -1.84127381 | 0.591635262 |
| TL - WK | -0.427401615 | 0.2097434 | -2.03773607 | 0.456144024 |
| TL - WT | -0.239954047 | 0.2096436 | -1.14458067 | 0.946917903 |
| WB - WK | -0.092443827 | 0.1815082 | -0.50930946 | 0.999620729 |
| WB - WT | 0.095003741 | 0.1813929 | 0.5237456 | 0.999543669 |
| WK - WT | 0.187447568 | 0.2092879 | 0.89564473 | 0.986537613 |

**Supplemental Table S3d.** Comparisons of estimated marginal means for PC1 for collective behavior across populations.

| **Comparison** | **Estimate** | **Standard error** | **t-ratio** | **p-value** |
| --- | --- | --- | --- | --- |
| FG - LG | -2.5547517 | 0.7534922 | -3.39054813 | 0.04485183 |
| FG - SL | 0.57994376 | 0.7040329 | 0.8237453 | 0.98971728 |
| FG - SR | -0.73956562 | 0.7534922 | -0.98151724 | 0.97247518 |
| FG - TL | -0.70890104 | 0.9637956 | -0.73553049 | 0.99475183 |
| FG - WB | -1.33633246 | 0.8295312 | -1.61094901 | 0.73927228 |
| FG - WK | -0.39154735 | 0.9637956 | -0.40625559 | 0.99988412 |
| FG - WT | 1.09060065 | 0.8296562 | 1.31452125 | 0.88372951 |
| LG - SL | 3.13469546 | 0.8063572 | 3.88747727 | 0.01510316 |
| LG - SR | 1.81518607 | 0.8498825 | 2.13580823 | 0.42427697 |
| LG - TL | 1.84585066 | 1.0408898 | 1.7733391 | 0.6428621 |
| LG - WB | 1.21841924 | 0.9179715 | 1.32729523 | 0.87869675 |
| LG - WK | 2.16320435 | 1.0408898 | 2.07822601 | 0.45733874 |
| LG - WT | 3.64535234 | 0.9180845 | 3.97060675 | 0.01251874 |
| SL - SR | -1.31950939 | 0.8063572 | -1.63638312 | 0.72469723 |
| SL - TL | -1.28884481 | 1.0056656 | -1.28158381 | 0.89616991 |
| SL - WB | -1.91627622 | 0.8778288 | -2.18297253 | 0.39802823 |
| SL - WK | -0.97149111 | 1.0056656 | -0.966018 | 0.9747511 |
| SL - WT | 0.51065688 | 0.8779469 | 0.58164892 | 0.99878224 |
| SR - TL | 0.03066458 | 1.0408898 | 0.02945997 | 1 |
| SR - WB | -0.59676683 | 0.9179715 | -0.65009296 | 0.99754246 |
| SR - WK | 0.34801828 | 1.0408898 | 0.33434689 | 0.99996884 |
| SR - WT | 1.83016627 | 0.9180845 | 1.99346177 | 0.50771351 |
| TL - WB | -0.62743142 | 1.0971887 | -0.57185368 | 0.99890777 |
| TL - WK | 0.31735369 | 1.2019164 | 0.26403974 | 0.99999379 |
| TL - WT | 1.79950169 | 1.0972832 | 1.63996101 | 0.72262846 |
| WB - WK | 0.94478511 | 1.0971887 | 0.86109626 | 0.98671889 |
| WB - WT | 2.4269331 | 0.9814533 | 2.47279537 | 0.2574284 |
| WK - WT | 1.48214799 | 1.0972832 | 1.35074334 | 0.86913296 |

**Supplemental Table S3e.** Comparisons of estimated marginal means for PC2 for collective behavior across populations.

| **Comparison** | **Estimate** | **Standard error** | **t-ratio** | **p-value** |
| --- | --- | --- | --- | --- |
| FG - WB | -0.82079167 | 0.2486062 | -3.30157404 | 0.082123836 |
| FG - WT | -1.27638542 | 0.2333555 | -5.46970473 | 0.003883757 |
| FG - TL | -0.98699307 | 0.2486062 | -3.97010695 | 0.02717755 |
| FG - LG | -0.64638396 | 0.3173423 | -2.03686645 | 0.491611643 |
| FG - SL | -1.22719644 | 0.2733781 | -4.48900864 | 0.007157727 |
| FG - SR | -1.74981124 | 0.3173423 | -5.51395461 | 0.001209587 |
| FG - WK | -0.9764676 | 0.2745047 | -3.55719874 | 0.058310947 |
| WB - WT | -0.45559375 | 0.2654101 | -1.71656549 | 0.678576161 |
| WB - TL | -0.1662014 | 0.2789135 | -0.59588863 | 0.998360398 |
| WB - LG | 0.17440771 | 0.3416049 | 0.51055391 | 0.99941446 |
| WB - SL | -0.40640478 | 0.3012031 | -1.34927166 | 0.866973874 |
| WB - SR | -0.92901958 | 0.3416049 | -2.7195735 | 0.187101412 |
| WB - WK | -0.15567593 | 0.302226 | -0.51509781 | 0.999330072 |
| WT - TL | 0.28939235 | 0.2654101 | 1.09035937 | 0.947563617 |
| WT - LG | 0.63000146 | 0.3306715 | 1.90521856 | 0.567634734 |
| WT - SL | 0.04918898 | 0.2887439 | 0.17035504 | 0.999999656 |
| WT - SR | -0.47342582 | 0.3306715 | -1.43171044 | 0.829359187 |
| WT - WK | 0.29991782 | 0.2898108 | 1.03487467 | 0.958882245 |
| TL - LG | 0.34060911 | 0.3416049 | 0.99708503 | 0.968163198 |
| TL - SL | -0.24020337 | 0.3012031 | -0.79747981 | 0.990910482 |
| TL - SR | -0.76281817 | 0.3416049 | -2.23304238 | 0.384143309 |
| TL - WK | 0.01052547 | 0.302226 | 0.03482649 | 1 |
| LG - SL | -0.58081248 | 0.3600339 | -1.61321599 | 0.737132796 |
| LG - SR | -1.10342728 | 0.3944554 | -2.79734392 | 0.161438053 |
| LG - WK | -0.33008364 | 0.3608901 | -0.91463754 | 0.979661045 |
| SL - SR | -0.5226148 | 0.3600339 | -1.45157099 | 0.821608871 |
| SL - WK | 0.25072884 | 0.3229103 | 0.77646588 | 0.992039678 |
| SR - WK | 0.77334364 | 0.3608901 | 2.14287847 | 0.4336955 |

**Supplemental Table S5.** Spearman's correlations between behavioral and morphological traits.

|  | **PC1-M** | **PC2-M** |
| --- | --- | --- |
| **PC1-CB** | 0.13  (p = 0.093) | -0.056  (p = 0.48) |
| **PC2-CB** | -0.04  (p = 0.59) | -0.000032  (p = 0.99) |
| **Boldness** | -0.00059  (p = 0.99) | **-0.22**  **(p < 0.01)** |
